# Supplementary material for: A scaffold attachment factor PHM-2 regulates synaptic transmission through SLO-2 potassium channel in C. elegans
Source: PLoS Genet. 2026 Mar 19;22(3):e1011962. doi: 10.1371/journal.pgen.1011962 (PMC13016476; doi:10.1371/journal.pgen.1011962)
Supplement: S1 Table — (DOCX) [file pgen.1011962.s002.docx]

*C. elegans* strains

| Strain name | Genotype | Source |
| --- | --- | --- |
| wild type | N2 Bristol | CGC |
| LY101 | *slo-2(nf101)* | CGC |
| ZW083 | *zwIs101[Pslo-1::slo-1::GFP]* | This study |
| ZW320 | *zwIs129[Pslo-1::slo-1(gf); pmyo-2::YFP]* | This study |
| ZW860 | *zwIs[Pslo-1::slo-2(gf), Pmyo-2::yfp]* | This study |
| ZW863 | *zwIs[Pslo-1::slo-2(gf), Pmyo-2::yfp];* *phm-2(zw67)*. ZW975: *zwEx221[Prab-3::slo-2::GFP]*; *phm-2(zw67)* | This study |
| ZW996 | *phm-2(zw67)* | This study |
| ZW1049 | *zwEx221[Prab-3::slo-2::GFP]* | This study |
| ZW1051 | *zwEx221[Prab-3::slo-2::GFP]; hrpu-2(zw97)* | This study |
| ZW1070 | *phm-2(zw93)* | This study |
| ZW1357 | *phm-2(zw93)*;*slo-2(nf101)* | This study |
| ZW1365 | *zwEx401[Prab-3::phm-2, Pmyo-2:: mStrawberry]; phm-2(zw93)* | This study |
| ZW1387 | *zwEx247[Pslo-2::mStrawberry, lin-15(+)]; zwEx403[Pphm-2::GFP, fosmid WRM064cE02, lin-15(+)]; lin-15(n765)* | This study |
| ZW1395 | *hrpu-2(zw97)* | This study |
| ZW1404 | *zwEx221[Prab-3::slo-2::GFP]*; *phm-2(zw93)* | This study |
| ZW1609 | *zwEx402[Prab-3::phm-2::GFP, Prab-3::His-58::mStrawberry, lin-15(+)]; lin-15(n765)* | This study |
| BJC201 | *zwIs101[Pslo-1::slo-1::GFP]*; *phm-2(zw93)* | This study |
| BJC272 | *phm-2(zw93); hrpu-2(zw97)* | This study |
| BJC273 | *zwEx221[Prab-3::slo-2::GFP]; phm-2(zw93);hrpu-2(zw97)* | This study |
| BJC284 | *phm-2(zw93); zwIs129[Pslo-1::slo-1(gf); pmyo-2::YFP]* | This study |
| BJC285 | *hrpu-2(zw97); zwIs129[Pslo-1::slo-1(gf); pmyo-2::YFP]* | This study |
| BJC287 | *phm-2(gk5114* *[loxP + myo-2p::GFP::unc-54 3' UTR + rps-27p::neoR::unc-54 3' UTR + loxP]); zwEx221[Prab-3::slo-2::GFP]* | This study |
